# Formulation-Driven Optimization of PEG-Lipid Content in Lipid Nanoparticles for Enhanced mRNA Delivery In Vitro and In Vivo

**DOI:** 10.3390/pharmaceutics17080950

**Published:** 2025-07-22

**Authors:** Wei Liu, Meihui Zhang, Huiyuan Lv, Chuanxu Yang

**Affiliations:** Key Laboratory of Colloid and Interface Chemistry of the Ministry of Education, School of Chemistry and Chemical Engineering, Shandong University, Jinan 250100, China; liuwei1992@sdu.edu.cn (W.L.); meihuizhang@mail.sdu.edu.cn (M.Z.); hylv@mail.sdu.edu.cn (H.L.)

**Keywords:** lipid nanoparticle, mRNA delivery, PEG-lipid content, formulation optimization, transfection efficiency

## Abstract

**Background**: Lipid nanoparticles (LNPs) represent one of the most effective non-viral vectors for nucleic acid delivery and have demonstrated clinical success in siRNA therapies and mRNA vaccines. While considerable research has focused on optimizing ionizable lipids and helper lipids, the impact of PEGylated lipid content on LNP-mediated mRNA delivery, especially in terms of in vitro transfection efficiency and in vivo performance, remains insufficiently understood. **Methods**: In this study, LNPs were formulated using a self-synthesized ionizable lipid and varying molar ratios of DMG-PEG2000. Nanoparticles were prepared via nanoprecipitation, and their physicochemical properties, mRNA encapsulation efficiency, cellular uptake, and transfection efficiency were evaluated in HeLa and DC2.4 cells. In vivo delivery efficiency and organ distribution were assessed in mice following intravenous administration. **Results**: The PEGylated lipid content exerted a significant influence on both the in vitro and in vivo performance of LNPs. A bell-shaped relationship between PEG content and transfection efficiency was observed: 1.5% DMG-PEG2000 yielded optimal mRNA transfection in vitro, while 5% DMG-PEG2000 resulted in the highest transgene expression in vivo. This discrepancy in optimal PEG content may be attributed to the trade-off between cellular uptake and systemic circulation: lower PEG levels enhance cellular internalization, whereas higher PEG levels improve stability and in vivo bioavailability at the expense of cellular entry. Furthermore, varying the PEG-lipid content enabled the partial modulation of organ distribution, offering a formulation-based strategy to influence biodistribution without altering the ionizable lipid structure. **Conclusions**: This study highlights the critical role of PEGylated lipid content in balancing nanoparticle stability, cellular uptake, and in vivo delivery performance. Our findings provide valuable mechanistic insights and suggest a straightforward formulation-based strategy to optimize LNP/mRNA systems for therapeutic applications.

## 1. Introduction

Messenger RNA (mRNA)-based therapeutics have emerged as a transformative modality, which holds great promise for use in the prevention and treatment of a wide range of diseases, ranging from infectious diseases to cancer and genetic disorders [1,2]. The unprecedented success of mRNA vaccines during the COVID-19 pandemic has underscored the clinical potential of this platform [3,4]. Compared with traditional attenuated or replicated vaccines, mRNA vaccines exhibit many advantages, including rapid and stable synthesis, a robust immune response, and favorable safety profiles [5,6].

The clinical translation of mRNA therapeutics critically depends on the development of safe and effective delivery systems. Among various non-viral vectors, lipid nanoparticles (LNPs) have proven to be the most efficient and clinically validated carriers for mRNA delivery. Notably, the LNP formulations used in the Pfizer-BioNTech (BNT162b2) and Moderna (mRNA-1273) COVID-19 vaccines were approved by both the U.S. FDA and the European Medicines Agency (EMA) in 2020, marking a major milestone in the field [7,8]. Since then, LNP/mRNA systems have been rapidly expanded to other therapeutic areas, including influenza, cancer immunotherapy, pulmonary diseases, and inflammatory conditions [6,9,10,11,12]. Given their pivotal role in enabling mRNA therapeutics, the continued advancement and compositional optimization of LNPs is essential for expanding their clinical utility across diverse disease indications.

A typical LNP is composed by four main components: ionizable lipids, helper lipids, cholesterol, and PEGylated lipids [13,14]. Each plays a distinct role in stabilizing the nanoparticle structure and facilitating intracellular delivery. Ionizable lipids condense mRNA through electrostatic interactions and mediate endosomal escape in acidic environments. Cholesterol reinforces membrane stability and fluidity, while helper lipids promote membrane fusion. [14,15,16]. PEGylated lipids, though present in relatively low molar ratios, are crucial for enhancing colloidal stability, prolonging circulation time, and modulating interactions with serum proteins and immune cells [17]. Despite their well-documented benefits, PEGylated lipids present a significant formulation challenge, often referred to as the “PEG dilemma”. While PEG moieties improve colloidal stability, reduce nonspecific uptake by the mononuclear phagocyte system, and prolong systemic circulation time, they can also impair cellular uptake and endosomal escape due to steric hindrance and the formation of a dense hydration shell [18,19,20].

To address these issues, recent studies have explored the structural optimization of PEG-lipids. For example, the FDA approved LNPs with DMG-PEG and ALC-PEG, which had a C14 lipid tail that could easily detach from the LNP surface to enhance cellular uptake and mRNA release. In contrast, DSPE-PEG with a long tail chain prolonged the circulation time of LNP in vivo, easily accumulated in the liver, and was more likely to induce an anti-PEG antibody response [21]. Zhang et al. systematically investigated how variations in lipid tail length and linkage type between PEG and lipid affected the in vitro and in vivo performance of mRNA-loaded LNPs; however, their study primarily focused on PEG-lipid structures and was restricted to subcutaneous administration [18]. In another study, Gao et al. demonstrated that modifying PEG terminal groups could enhance liver-targeted mRNA delivery. Nevertheless, their work employed a fixed PEG-lipid content (1.5%) and did not explore the consequences of varying PEG-lipid concentration [22]. Although such studies have begun to elucidate the role of PEG-lipid chemistry, comprehensive investigations evaluating the impact of PEG-lipid content on both in vitro and in vivo LNP performance remain scarce. This lack of systematic data limits the translational relevance of in vitro screening assays, which are often used to predict in vivo outcomes, and poses a barrier to the rational and efficient design of LNPs for clinical mRNA delivery.

In this study, we employed a synthetic ionizable lipid to formulate LNPs with varying contents of DMG-PEG2000, a clinically used short-chain PEG-lipid. Using a nanoprecipitation method, we prepared LNPs and characterized their physicochemical properties. We then evaluated their mRNA delivery efficiency in vitro using HeLa and DC2.4 dendritic cells and in vivo following systemic administration in mice. Particular emphasis was placed on comparing the PEG-lipid content-dependent transfection efficiency between in vitro and in vivo settings. Furthermore, we investigated whether simple modulation of PEG-lipid content could affect organ-level biodistribution. This work aims to provide mechanistic insights and formulation-based strategies to optimize LNPs for mRNA delivery, ultimately contributing to the rational development of next-generation RNA therapeutics.

## 2. Materials and Methods

### 2.1. Materials

All reagents used in this study were of analytical grade unless otherwise stated. Tris(2-aminoethyl) amine, tetradecyl acrylate, D-fluorescein potassium salt, 1,2-dimyristoyl-rac-glycero-3-methoxypolyethylene glycol (DMG-PEG), and 1,2-dioleoyl-sn-glycero-3-phosphoethanolamine (DOPE) were obtained from Aladdin (Shanghai, China). Cholesterol was purchased from Sigma-Aldrich (St. Louis, MO, USA). Wheat germ agglutinin Alexa Fluor 488 conjugate (WGA-AF488) and anti-fade mounting medium containing DAPI were purchased from Thermo Fisher Scientific (Waltham, MA, USA). The mRNAs encoding firefly luciferase (mLuc), green fluorescent protein (mEGFP), Cy5-labeled mEGFP, and Cell Counting Kit-8 (CCK-8) were purchased from APExBIO (Huston, TX, USA).

### 2.2. Synthesis of Ionizable Lipid

**Figure 1:**
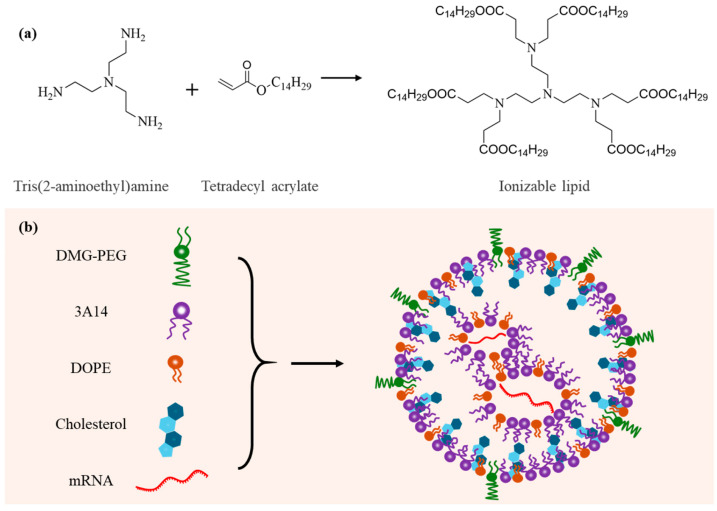
Schematic illustration of the design of LNPs for mRNA delivery. (**a**) Chemical synthesis route and molecular structure of the ionizable lipid. (**b**) Composition and structural model of the lipid nanoparticle (LNP).

The ionizable lipid was synthesized via a Michael addition reaction between tris(2-aminoethyl)amine and tetradecyl acrylate according to previous reports [23,24]. The synthetic route is illustrated in Figure 1a. Briefly, tris(2-aminoethyl)amine (87.74 mg, ~0.6 mmol) and tetradecyl acrylate (804 mg, ~3 mmol) were added to a 5 mL reaction vial. The mixture was stirred magnetically at 90 °C for 72 h under light-protected conditions. Upon completion, the crude product was purified via column chromatography to yield the desired compound, which was stored at −20 °C in the dark for further use. A small amount of the product was dissolved in deuterated chloroform (CDCl_3_) and characterized by H^1^-NMR spectroscopy (Figure S1).

### 2.3. Synthesis and Characterization of LNP

Lipid nanoparticles (LNPs) were synthesized using a nanoprecipitation method [25,26]. In brief, ionizable lipid, cholesterol, DMG-PEG, and DOPE were each dissolved in anhydrous ethanol at final concentrations of 100, 25, 50, and 25 mg/mL, respectively. These components were then mixed at a molar ratio of ionizable lipid/cholesterol/DMG-PEG/DOPE = 40:(50−X):X:10, where X ranged from 0.1 to 10. Under continuous vortexing, the organic phase was added dropwise into acetate buffer (200 mM, pH 5.4) to facilitate the self-assembly of LNPs. The resulting nanoparticles were dialyzed and stored at 4 °C for further use. Based on our previous optimization studies [23,25], LNPs and mRNA were mixed at an N/P ratio of 12.5:1 for efficient encapsulation.

The average hydrodynamic diameter, polydispersity index (PDI), and zeta potential of the LNP/mRNA complexes were measured by dynamic light scattering (DLS) using a ZetaSizer NanoZS (Malvern Instruments, Worcestershire, UK). Transmission electron microscopy (TEM) was used to observe the morphology of LNP/mRNA particles after negative staining with 1% uranyl acetate.

The encapsulation efficiency of mRNA within the LNPs was quantified using the Quant-iT™ RiboGreen RNA Assay Kit (Thermo Fisher Scientific). Briefly, RiboGreen stock solution was diluted 1:200 with 1× TE buffer (pH 7.5) and 50 μL was added per well in a black 96-well plate. LNP/mRNA complexes containing 0.1 μg of mRNA were diluted with TE buffer to a final concentration of 0.4 μg/mL, and 50 μL of this dilution was added to the wells containing RiboGreen. To calculate the encapsulation efficiency, free mRNA not formulated with LNPs and at the same concentration was included as total mRNA content, and TE buffer alone served as the negative control. After incubation at room temperature for 10 min, fluorescence was measured using a multimode microplate reader (excitation: 480 nm; emission: 520 nm).

### 2.4. Cell Toxicity Analysis

HeLa cells and DC 2.4 cells were cultured at 37 °C in a humidified incubator with 5% CO_2_. HeLa cells were maintained in DMEM complete medium supplemented with 10% fetal bovine serum (FBS) and 1% penicillin–streptomycin (P/S), while DC 2.4 cells were cultured in RPMI-1640 complete medium with the same supplements.

The cytotoxicity of LNP/mRNA complexes was evaluated in HeLa cells using the Cell Counting Kit-8 (CCK-8) assay. Briefly, HeLa cells were seeded in 96-well plates at a density of 10,000 cells per well and incubated for 24 h. The culture medium was then replaced with fresh medium containing LNP/mRNA complexes at varying mRNA concentrations. After 24 h of incubation, the medium was removed, and CCK-8 reagent diluted 1:10 (*v*/*v*) in fresh medium was added to each well. After 30 min of incubation, absorbance at 450 nm was measured using a multifunctional microplate reader. Untreated cells served as the control group, and wells containing only CCK-8 working solution were used as the blank group.

### 2.5. Cellular Uptake and In Vitro Transfection Analysis

The cellular uptake of LNP/mRNA complexes was analyzed using both flow cytometry and confocal laser scanning microscopy (CLSM). For flow cytometry, HeLa cells were seeded in 24-well plates (50,000 cells/well) and incubated overnight. The medium was replaced with fresh medium containing LNPs loaded with Cy5-labeled mRNA (final RNA concentration: 0.4 μg/mL). After 6 h of incubation, intracellular Cy5 fluorescence intensity was quantified by flow cytometry. For confocal imaging, transfected cells were washed three times with 1× PBS, fixed with 4% paraformaldehyde (PFA) for 15 min, and washed again with PBS. Membranes were stained with WGA-AF488 (2 μg/mL) for 10 min, followed by three PBS washes. Nuclei were stained using DAPI-containing anti-fade mounting medium. Cellular internalization was visualized using confocal laser scanning microscopy.

To assess mRNA transfection efficiency, HeLa or DC 2.4 cells were seeded in 24-well plates at a density of 50,000 cells per well and incubated overnight. The medium was then replaced with fresh medium containing LNP/mEGFP complexes (final mEGFP concentration: 0.4 μg/mL). Cells without LNP/mEGFP treatment served as the blank control group. After 24 h of incubation, green fluorescent protein (EGFP) expression was observed using a fluorescence microscope, and transfection efficiency was quantified by flow cytometry.

### 2.6. In Vivo mRNA Delivery Assessment

All animal experiments were performed in accordance with the Health Guide for the Care and Use of Laboratory Animals of National Institutes. Female BALB/c mice (6–8 weeks old) were purchased from Jinan Pengyue Experimental Animal Co., Ltd. (Jinan, China).

To evaluate the in vivo mRNA delivery efficiency of the LNPs, Luc mRNA LNPs were administered to BALB/c mice via tail vein injection at an mRNA dose of 0.2 mg/kg. After 6 h, D-luciferin was injected subcutaneously at a dose of 200 mg/kg. Ten minutes post-injection, the mice were euthanized, and major organs including the heart, liver, spleen, lungs, and kidneys were harvested. Bioluminescence imaging was then performed using a small animal in vivo imaging system, and luciferase expression was quantified by measuring the luminescent signal intensity in each organ.

### 2.7. In Vivo Safety Evaluation

To evaluate the biosafety of the LNPs in vivo, BALB/c mice were intravenously injected with LNP/mRNA complexes at an mRNA dose of 0.2 mg/kg. After 24 h, the mice were euthanized, and major organs, including the heart, liver, spleen, lungs, and kidneys, were harvested and fixed in 4% paraformaldehyde (PFA). The fixed tissues were subsequently embedded in paraffin, sectioned, and stained with hematoxylin and eosin (H&E) for histological analysis. Tissue sections were examined under a light microscope. Untreated mice served as the control group.

### 2.8. Statistics

All experimental results are presented as the mean ± standard deviation (SD) from at least three independent experiments. The unpaired student’s *t*-test was used to assess statistically significant differences between groups (* *p* < 0.05, ** *p* < 0.01, and *** *p* < 0.001).

## 3. Results

### 3.1. Synthesis and Characterization of LNP/mRNA Formulations with Varying DMG-PEG Content

**Figure 2:**
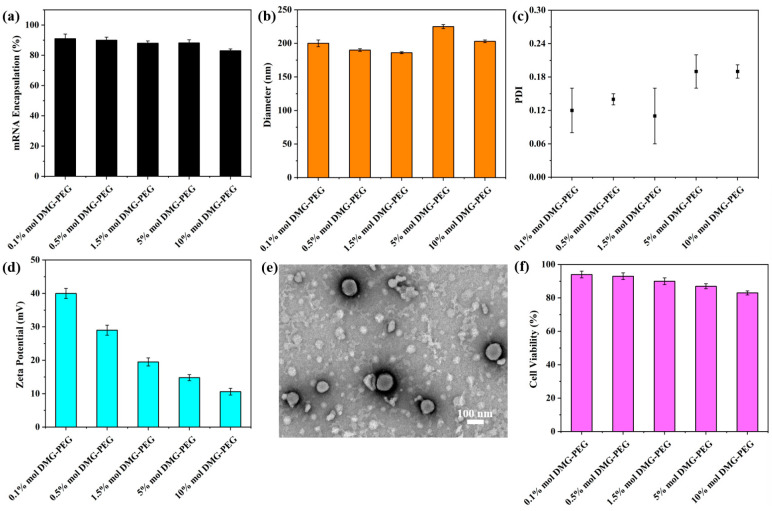
Physicochemical and biological characterization of LNPs. (**a**) Encapsulation efficiency of mRNA in LNPs formulated with varying DMG-PEG contents. (**b**) Hydrodynamic diameter of different LNP formulations by DLS. (**c**) Polydispersity index (PDI) distribution of LNPs with varying particle sizes. (**d**) Zeta potential of different LNPs. The error bars represent the standard deviation of repeat DLS measurements (*n* = 3). (**e**) Representative TEM image of LNPs synthesized with 1.5 mol% DMG-PEG. (**f**) Cytotoxicity of LNPs on HeLa cells at a lipid dose of 8 μg/mL, evaluated by CCK-8 assay (*n* = 4).

The ionizable lipid was synthesized via a Michael addition reaction (Figure 1a). Lipid nanoparticles (LNPs) containing varying molar ratios of DMG-PEG were subsequently formulated using a nanoprecipitation method (Figure 1b). Due to the strong hydration effect of PEG chains, an excessive amount of DMG-PEG may shield the positive charge of the ionizable lipid, thereby weakening the electrostatic interaction with mRNA and potentially impairing the mRNA-loading capacity of the nanoparticles. To evaluate this effect, we first characterized the mRNA encapsulation efficiency of LNPs with varying DMG-PEG contents. The results showed that all LNP formulations exhibited encapsulation efficiencies greater than 80%, indicating efficient mRNA loading across all tested groups (Figure 2a). These findings suggest that the mRNA-loading capacity was not significantly affected by DMG-PEG content within the tested range, up to 10%.

Next, we specifically evaluated the impact of varying DMG-PEG content on particle size and zeta potential. It has been pointed out that the particle size of LNP depends largely on the components of LNP [27], the preparation method [26,28], and the affinity between the PEGylated lipids and the lipophilic core of LNP [22]. Dynamic light scattering (DLS) was employed to measure both particle size and polydispersity index (PDI). As shown in Figure 2b,c, the DMG-PEG content had only a minor effect on particle size. All LNP formulations exhibited a narrow size distribution, with particle diameters ranging from 180 to 230 nm. Notably, LNPs containing 5% and 10% DMG-PEG showed slightly increased diameters (>200 nm), whereas those with 0.1–1.5% DMG-PEG maintained sizes below 200 nm. Despite these slight differences, the overall impact of DMG-PEG content on particle size remained limited. Transmission electron microscopy (TEM) images further revealed that all of the LNP/mRNA exhibited a uniform spherical morphology (Figures 2e and S2), indicating successful nanoparticle self-assembly. Moreover, the zeta potential measurements revealed that all LNP formulations carried a positive surface charge (Figure 2d), which may facilitate electrostatic interactions with the negatively charged cell membrane, thereby enhancing cellular uptake. With increasing DMG-PEG content, the zeta potential exhibited a decreasing trend, likely due to enhanced surface shielding by the PEG chains.

We further evaluated the in vitro biocompatibility of the LNP formulations using HeLa cells. As shown in Figure 2f, all LNPs exhibited high cell viability, confirming their good biocompatibility. However, a slight decrease in cell viability was observed with increasing DMG-PEG content, suggesting that higher molar ratios of DMG-PEG may pose a potential cytotoxicity risk. These findings highlight the importance of optimizing PEG-lipid content to balance formulation stability and biocompatibility.

### 3.2. Effect of DMG-PEG Content on In Vitro Transfection Efficiency of LNP/mRNA

**Figure 3:**
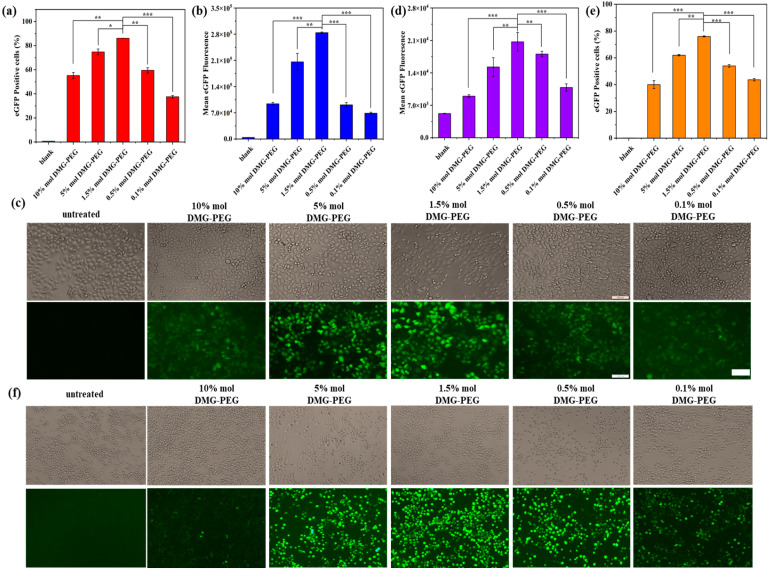
Transfection performance of LNP/mEGFP complexes in HeLa and DC 2.4 cells. (**a**) Transfection efficiency of LNP/mEGFP in HeLa cells measured by flow cytometry. (**b**) Quantitative analysis of EGFP fluorescence intensity in transfected HeLa cells. (**c**) Representative fluorescence images of EGFP expression in HeLa cells after transfection. (**d**) Quantitative analysis of EGFP fluorescence intensity in DC 2.4 cells. (**e**) Transfection efficiency of LNP/mEGFP in DC 2.4 cells quantified by flow cytometry. (**f**) Representative fluorescence images of EGFP expression in DC 2.4 cells; scale bars: 100 μm. Date are shown as mean ± SD of three repetitions. (* *p* < 0.5, ** *p* < 0.01, *** *p* < 0.001).

The in vitro transfection efficiency of LNP/mRNA formulations with varying DMG-PEG content was evaluated in both HeLa cells and DC 2.4 dendritic cells (Figure 3). As shown in Figures 3a–c and S3a, transfection efficiency in HeLa cells was significantly influenced by the DMG-PEG molar ratio in the LNP formulation. Specifically, transfection efficiency exhibited a biphasic trend: it initially increased with higher DMG-PEG content, peaking at 1.5%, and subsequently decreased as the PEG content continued to rise. At 1.5% and 5% DMG-PEG, the fluorescence intensity of mEGFP expression in HeLa cells was 3.1-fold and 2.3-fold higher, respectively, compared to the LNPs containing 10% DMG-PEG. A similar trend was observed in DC 2.4 cells (Figures 3d–f and S3b), where transfection efficiency was also maximized at a DMG-PEG content of 1.5%. Relative to the 10% DMG-PEG group, fluorescence intensity increased 2.3-fold and 1.67-fold in the 1.5% and 5% DMG-PEG groups, respectively. These results suggest that a moderate amount of DMG-PEG (approximately 1.5%) optimizes LNP-mediated mRNA delivery across different cell types.

To further substantiate our conclusion that 1.5 mol% DMG-PEG offers optimal in vitro transfection efficiency, we performed a kinetic study in which HeLa cells were transfected with LNPs containing different DMG-PEG contents. Luciferase expression was measured at multiple time points (3, 6, 12, and 24 h) using a luciferase assay. As shown in Figure S4, luciferase expression progressively increased over time for most formulations. Notably, the LNPs with 1.5 mol% DMG-PEG consistently yielded the highest luciferase levels at all time points. These findings provide compelling kinetic evidence that supports 1.5 mol% DMG-PEG as the optimal formulation for in vitro mRNA delivery. Moreover, this kinetic analysis offers valuable complementary data and strengthens the effect of PEG-lipid content on modulating transfection performance over time.

**Figure 4:**
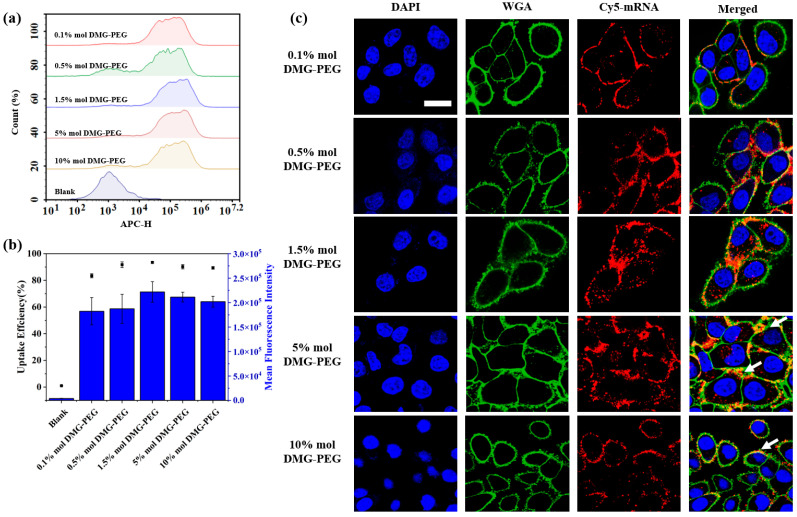
Effect of DMG-PEG content on cellular uptake of LNP/mRNA in HeLa cells. (**a**) Flow cytometry histogram showing fluorescence intensity of HeLa cells treated with LNP/mRNA containing different DMG-PEG contents. (**b**) Quantitative analysis of cellular uptake, including the percentage of Cy5-positive cells (black dots) and mean fluorescence intensity (blue bars) based on flow cytometry results (*n* = 3). (**c**) Representative confocal fluorescence images of HeLa cells after incubation with LNP/Cy5-mRNA; scale bars: 20 μm. White arrows indicate LNP accumulation on the cell surface.

To further investigate the underlying mechanism, we assessed the cellular uptake of LNPs loaded with Cy5-labeled mRNA in HeLa cells using flow cytometry and confocal microscopy (Figure 4). The cellular uptake profile from flow cytometry analysis generally mirrored the transfection results, with the highest uptake observed at 1.5% DMG-PEG. Interestingly, although the transfection efficiency decreased at higher DMG-PEG levels (≥5%), the cellular uptake and associated fluorescence intensity remained relatively high, surpassing that of LNPs with lower PEG content (≤0.5%). This phenomenon may be attributed to increased LNP accumulation on the cell surface at higher PEG levels, as indicated by the enhanced peripheral fluorescence signals (highlighted in the white arrows, Figure 4c). Excessive PEGylation is known to hinder intracellular trafficking by impeding cell endocytosis and endosomal escape, ultimately reducing mRNA translation efficiency despite sufficient uptake [18,29]. Confocal fluorescent cell imaging further confirmed that the strongest intracellular fluorescence signal was observed in the 1.5% DMG-PEG group. These findings underscore the importance of fine-tuning PEG content in LNP formulations to balance intracellular uptake and transfection efficiency.

### 3.3. Influence of DMG-PEG Content on In Vivo Biodistribution and Transfection Efficiency of LNP/mRNA in Mice

**Figure 5:**
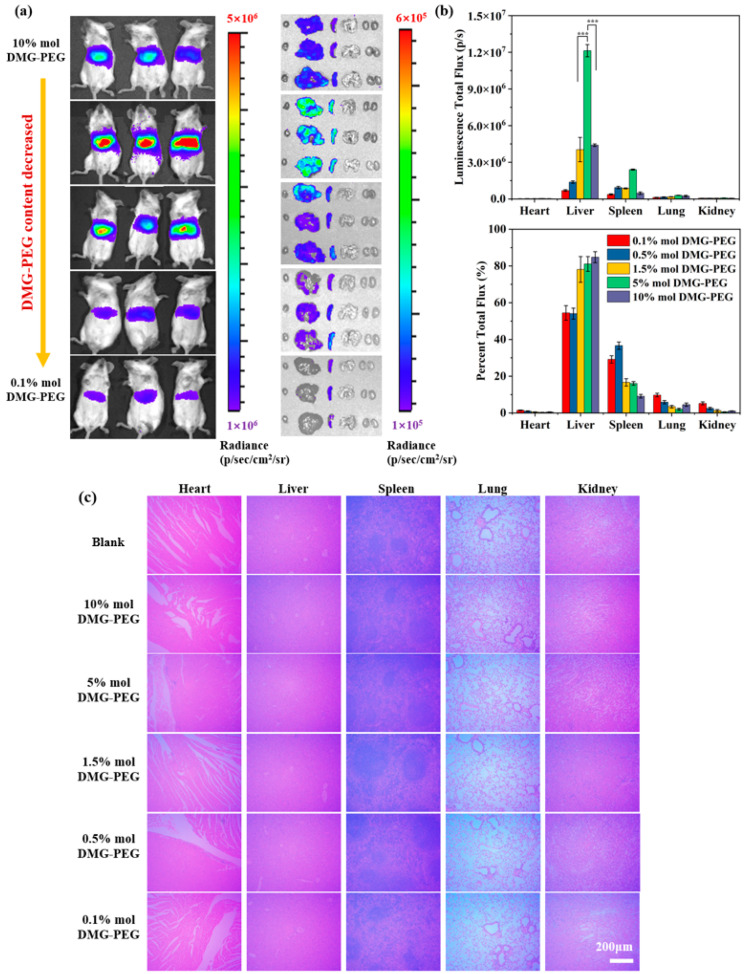
In vivo transfection efficiency and biosafety evaluation of Luc mRNA LNPs in mice. (**a**) In vivo bioluminescence imaging of mice and ex vivo bioluminescence imaging of major organs (heart, liver, spleen, lung, and kidneys) 6 h after intravenous injection of Luc mRNA LNPs via tail vein. (**b**) Quantitative analysis and the percentage of luciferase expression in different organs based on bioluminescence intensity (*n* = 3, *** *p* < 0.001). (**c**) Hematoxylin and eosin (H&E) staining of major organs 24 h post-injection for histopathological evaluation; scale bars: 200 μm.

To investigate the effect of DMG-PEG content on in vivo transfection, LNPs encapsulating mLuc mRNA were intravenously administered to mice. As shown in Figure 5, bioluminescence imaging at 6 h post-injection revealed that the fluorescent signals were predominantly localized in the liver and spleen. Among all tested groups, LNPs containing 5% DMG-PEG produced the highest bioluminescent signals in both organs. Specifically, liver signal intensities in the 5% DMG-PEG group were 3.2-fold and 2.7-fold higher than those observed in the 1.5% and 10% DMG-PEG groups, respectively. Similarly, the spleen signals were 3.3- and 5.0-fold higher than those in the 1.5% and 10% groups. For a clearer comparative analysis, taking the 10% DMG-PEG group as a reference, liver fluorescence intensities in the 1.5% and 5% groups decreased by 1.2-fold and increased by 2.6-fold, respectively. In the spleen, fluorescence intensities increased by 1.7-fold and 5.4-fold in the 1.5% and 5% groups, respectively. These results indicate that a fine-tuning of DMG-PEG content, specifically to 5%, markedly enhances LNP-mediated mRNA delivery to key target organs in vivo.

To further support our conclusion that 5 mol% DMG-PEG yields optimal in vivo transfection efficiency, we conducted a kinetic study in which mice were intravenously injected with LNPs containing varying DMG-PEG contents. Luciferase expression was monitored at multiple time points (3, 6, 12, and 24 h) using an IVIS bioimaging system. As shown in Figure S5, luciferase expression peaked at 6 h post-injection across all formulations. Importantly, the formulation containing 5 mol% DMG-PEG consistently exhibited the highest expression at each time point, reinforcing our conclusion that 5 mol% represents the optimal PEG-lipid content for in vivo mRNA delivery. It is worth noting that, in contrast, the in vitro kinetic profile showed a continuous increase in luciferase expression from 3 to 24 h post-transfection for most LNP formulations. This discrepancy likely arises from fundamental differences between in vitro and in vivo environments, including factors such as cellular uptake dynamics, bioavailability, and degradation pathways. In vivo systems are influenced by additional complexities, such as blood circulation, tissue-specific distribution, immune interactions, and metabolic clearance, all of which can significantly impact the kinetics of mRNA expression. These observations underscore the importance of evaluating LNP performance in both in vitro and in vivo settings to fully understand the impact of PEG-lipid composition on delivery efficiency.

Interestingly, the optimal DMG-PEG content for in vivo transfection (5%) was higher than that observed for in vitro transfection (1.5%). This discrepancy may result from physiological barriers in the bloodstream. LNPs with insufficient PEGylation (e.g., 1.5% DMG-PEG) may be rapidly cleared by the mononuclear phagocyte system (MPS), limiting their circulation and bioavailability. Previous studies have shown that upon incubation with 40% mouse serum, approximately 40% and 12% of DMG-PEG molecules remained on LNP surfaces at 2 and 9 h, respectively, indicating that high serum protein concentrations accelerate PEG desorption [22]. Such de-PEGylation can enhance organ accumulation and mRNA delivery efficiency. However, excessively high PEG content (e.g., 10%) may stabilize LNPs too strongly, impeding PEG shedding, cellular uptake, and subsequent transfection, as observed from in vitro cell study. Notably, LNPs with 0.5% DMG-PEG content exhibited preferential accumulation in the spleen, where bioluminescence accounted for over 60% of the total body signal. These findings suggest that simple modulation of the four-component formulation of LNPs can enable partial organ-specific distribution. In our study, enhanced delivery to extrahepatic organs, particularly the spleen, was observed when the PEG-lipid content was reduced to 0.5% or 0.1%. Although the underlying mechanisms remain to be fully elucidated, such tissue-specific distribution may help minimize off-target accumulation and expand the therapeutic potential of mRNA-based treatments.

To further reinforce our conclusion that DMG-PEG content plays a critical role in modulating in vivo LNP performance, we additionally evaluated a standard Pfizer-LNP formulation consisting of the ionizable lipid ALC-0315 (50%) and the phospholipid DSPC (10%). As shown in Figure S6, the trend observed was consistent with our synthetic LNPs: ALC-0315-based LNPs containing 5% DMG-PEG exhibited the highest mRNA delivery efficiency in mice. It is noteworthy that while 5% DMG-PEG resulted in optimal in vivo transfection in both our synthetic LNPs and the Pfizer-LNP system, the ideal PEG-lipid content may vary depending on the specific ionizable lipids and co-lipids employed in different formulations. Therefore, further studies are warranted to systematically explore and optimize PEG content across diverse LNP compositions.

To assess biosafety, a histological examination of major organs was performed after LNP administration (Figure 5c). No obvious pathological abnormalities or tissue damage were observed in any group, indicating that all LNP formulations exhibited good biocompatibility. Furthermore, altering the DMG-PEG content did not induce observable toxicity, supporting the safety of PEG-lipid modulation in vivo.

## 4. Discussion

Lipid nanoparticles (LNPs) are among the most promising carriers for mRNA delivery and have already demonstrated their value in clinical applications. However, the LNP formulations still need to be fine-tuned to further enhance their therapeutic efficacy for various diseases. Typically, LNPs are composed of four key components: ionizable lipids, helper lipids, cholesterol, and PEGylated lipids. While extensive research has focused on optimizing the ionizable lipid structure or helper lipids to enhance mRNA delivery to the cytosol or achieve organ-specific targeting [30,31], there remains a lack of comprehensive studies examining the impact of PEG content in LNP formulations on mRNA delivery efficiency. PEG-lipids play a vital role in preventing aggregation and prolonging blood circulation time by reducing uptake by the reticuloendothelial system (RES). However, they also hinder cell uptake and endosome escape due to the hydration shell created by the PEG moiety, a phenomenon referred to as the “PEG dilemma”. Recent studies have begun to address aspects of PEGylation. For example, Zhang, et al. investigated how lipid tail length, and the chemical linkage between PEG and lipid influence the in vitro and in vivo behavior of mRNA-loaded LNPs, though their work primarily focused on PEG-lipid types and was limited to subcutaneous administration routes [18]. In another recent work, Gao et al. demonstrated that modifying PEG terminal groups can enhance liver-specific mRNA delivery; however, their study maintained a fixed PEG-lipid content of 1.5%, and did not evaluate the impact of varying PEG concentrations [22]. In addition, the rational design of stimuli-responsive degradable PEG holds great promise for targeted drug delivery in cancer therapy [32,33], which may inform the development of next-generation smart LNP systems. In this study, we systematically investigated the effect of PEGylated lipid content on the physicochemical properties, in vitro transfection efficiency, and in vivo biodistribution of LNP/mRNA formulations administered via intravenous injection. This work aims to provide practical guidance for optimizing PEG-lipid content to balance stability, circulation, and delivery efficiency in mRNA-based therapeutics.

Given that PEG-lipids with longer acyl chains are associated with increased safety concerns and reduced delivery efficiency [34], we selected DMG-PEG, a short-chain PEG-lipid, as the PEGylated component. Among various PEG molecular weights, PEG with a molecular weight of 2 kDa has been reported to offer an optimal balance between delivery efficiency and immunogenicity [34,35]. Accordingly, DMG-PEG2000 was employed in our formulations. To achieve LNPs with varying DMG-PEG content, the PEG-lipid proportion was adjusted by correspondingly reducing the cholesterol content, due to the relatively low overall PEG-lipid molar ratio in comparison to cholesterol. Cholesterol plays a crucial role in stabilizing the lipid bilayer by enhancing membrane rigidity and structural integrity [36,37]. However, considering the relatively low PEG content in comparison to cholesterol, slight alterations in cholesterol content may not significantly impact the overall formulation, as further confirmed by DLS characterization. Indeed, in many studies exploring LNP composition, the modulation of auxiliary components, such as helper lipids and PEG-lipids, is commonly achieved by adjusting the cholesterol content, thereby maintaining the ionizable lipid molar ratio constant [1,18,38].

There are several factors influencing the particle size of LNPs, including changes in LNP components, the type of ionizable lipids, and the preparation method [26,39]. In this study, LNPs were synthesized using the nanoprecipitation method and the obtained particle size was generally around 150–200 nm, which was basically consistent with the literature [26]. Interestingly, no significant correlation was observed between the alteration of PEGylated lipid content and changes in LNP particle size. The encapsulation efficiency of LNPs for mRNA remained largely unaffected with increasing DMG-PEG content up to 5%. However, at 10% DMG-PEG content, the encapsulation efficiency was the lowest. This may be due to the disruption of electrostatic interactions between mRNA molecules and ionizable lipids, caused by the charge shielding effect associated with higher DMG-PEG content. In line with this, the zeta potential of LNPs decreased as the DMG-PEG content increased.

The content of PEGylated lipids in LNP had a significant effect on the in vitro transfection of mRNA. In vitro transfection studies used two different types of cell lines, including a cancer cell line, HeLa, and dendritic cell line, DC2.4, which revealed a bell-shaped relationship between PEG-lipid content and transfection efficiency, with the optimal DMG-PEG content being 1.5% (Figure 3). This pattern likely reflects the dual role of PEG: while moderate PEGylation enhances nanoparticle stability, excessive PEGylation impedes cellular uptake and endocytosis, thus diminishing mRNA delivery efficacy [29,40,41,42]. Supporting this, our cellular uptake experiments (Figure 4) demonstrated a reduced internalization of highly PEGylated LNPs.

Moreover, the influence of PEG-lipid content on in vivo LNP/mRNA performance was even more pronounced. Bioluminescence imaging showed that transfection efficiency increased and then decreased with rising DMG-PEG content, peaking at 5% (Figure 5). This shift in optimal PEG-lipid content from 1.5% (in vitro) to 5% (in vivo) can be attributed to pharmacokinetic factors. After systemic administration, LNPs with low PEG content (<1.5%) may undergo rapid de-PEGylation and be prematurely cleared by mononuclear phagocyte systems, limiting their bioavailability and reducing delivery to target tissues. Conversely, LNPs with high PEG content (>5%) may suffer from poor cellular uptake and endocytosis due to steric hindrance from the dense PEG corona, resulting in reduced transfection efficiency. While this study focused on elucidating the impact of PEGylated lipid content using DMG-PEG2000, future investigations could benefit from a broader evaluation of PEG-lipids with varying molecular weights and structural features. PEG chain length is known to influence nanoparticle stability, circulation time, and cellular uptake behavior [43]. In future, a systematic comparison of PEG-lipids with different molecular weights, such as PEG1000, PEG2000, and PEG5000, could provide deeper insights into structure–activity relationships and help refine LNP design for specific therapeutic applications.

In this study, our primary objective was to investigate how varying PEG-lipid content influences the in vivo performance of mRNA-loaded lipid nanoparticles (LNPs), with a particular focus on systemic delivery and organ biodistribution. We therefore selected intravenous (tail vein) injection as the administration route, as it enables a comprehensive evaluation of pharmacokinetics, organ-specific distribution, and transgene expression across major organs such as the liver, spleen, and lungs. This route is especially relevant for therapeutic applications including gene therapy, protein replacement, and systemic vaccination. Notably, recent studies have suggested that spleen-targeted LNPs can enhance the efficacy of systemic mRNA vaccination [44,45]. Interestingly, our results showed that the LNP formulation containing 0.5% PEG-lipid exhibited enhanced accumulation in the spleen compared to other formulations, which may offer a promising avenue for future vaccine development. Beyond vaccination, the findings of this study may also provide valuable insights for optimizing LNP formulations in other mRNA-based therapeutic applications, such as in vivo gene therapy and genome editing. It is worth noting that intramuscular (IM) injection remains a clinically important route for mRNA vaccines, where local expression and antigen presentation at the injection site and draining lymph nodes are key to immune activation. While PEG-lipid content may also influence LNP behavior following IM administration, the underlying distribution patterns, tissue retention, and immune cell interactions might differ significantly from those in systemic circulation. Further studies are warranted to investigate how PEG-lipid content modulates LNP performance in the IM context.

In summary, our study provides valuable insights into the role of PEGylated lipid content in LNP-mediated mRNA delivery. By fine-tuning the DMG-PEG content, we were able to optimize both in vitro and in vivo transfection efficiency, and demonstrate the feasibility of organ-targeted delivery through simple compositional adjustments. These findings have important implications for the rational design of next-generation LNPs for therapeutic mRNA applications, such as mRNA vaccines or protein replacement therapy.

## 5. Conclusions

In this study, we systematically evaluated the impact of PEGylated lipid content on the physicochemical properties and delivery performance of lipid nanoparticles (LNPs) encapsulating mRNA, using DMG-PEG2000 as a representative short-chain PEG-lipid. Our findings provide important mechanistic insights and formulation strategies to optimize mRNA-LNP delivery systems. Key conclusions include the following: (1) PEG-lipid content critically modulates mRNA delivery efficiency, with significant effects observed both in vitro and in vivo. (2) Optimal PEG-lipid content differs between in vitro and in vivo settings, with 1.5% DMG-PEG yielding the highest transfection efficiency in vitro, and 5% achieving the best performance in vivo after systemic administration. (3) Lower PEG densities favor enhanced cellular uptake and endosomal escape, likely due to reduced steric hindrance and charge shielding, thus improving intracellular delivery. (4) Tuning PEG-lipid content enables partial organ-specific distribution of LNPs, representing a practical strategy for extrahepatic targeting through compositional optimization without altering the ionizable lipid structure. Together, these results highlight the dual role of PEG-lipids in balancing nanoparticle stability and delivery efficacy, and underscore the importance of rational PEGylation tuning in LNP design. This work establishes a formulation-based framework for improving the therapeutic potential of LNP/mRNA systems and provides practical guidance for developing next-generation mRNA therapeutics, including vaccines, gene therapies, and protein replacement treatments.

## Data Availability

The data presented in this study are available in this article.

## Supplementary Materials

The following supporting information can be downloaded at https://www.mdpi.com/article/10.3390/pharmaceutics17080950/s1, Figure S1: The H1 NMR spectra of ionizable lipid; Figure S2: Representative TEM images of all formulations with different DMG-PEG contents from 0.1% to 10%; Figure S3: Flow cytometry histograms showing fluorescence intensity of LNP/mRNA-transfected cells formulated with varying DMG-PEG contents; Figure S4: mRNA expression kinetics in vitro; Figure S5: mRNA expression kinetics in vivo; and Figure S6: In vivo transfection efficiency of ALC-0315-LNP/mluc in mice.
