# Supplementary material for: Formulation-Driven Optimization of PEG-Lipid Content in Lipid Nanoparticles for Enhanced mRNA Delivery In Vitro and In Vivo
Source: Pharmaceutics. 2025 Jul 22;17(8):950. doi: 10.3390/pharmaceutics17080950 (PMC12388858; doi:10.3390/pharmaceutics17080950)
Supplement: Supplementary file 1 [file pharmaceutics-17-00950-s001.zip › pharmaceutics-3726248-supplementary.pdf]

# Supporting Information

**Formulation-Driven Optimization of PEG-Lipid Content in Lipid Nanoparticles for Enhanced mRNA Delivery In Vitro and In Vivo**

Wei Liu, Meihui Zhang, Huiyuan Lv, Chuanxu Yang*

Key Laboratory of Colloid and Interface Chemistry of the Ministry of Education, and School of Chemistry and Chemical Engineering, Shandong University, Jinan 250100, China

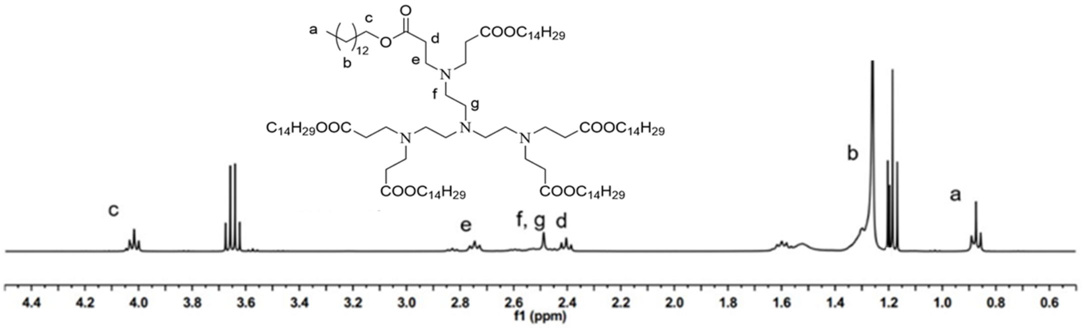


**Figure S1.** The $H^1$ NMR spectra of the synthetic ionizable lipid.

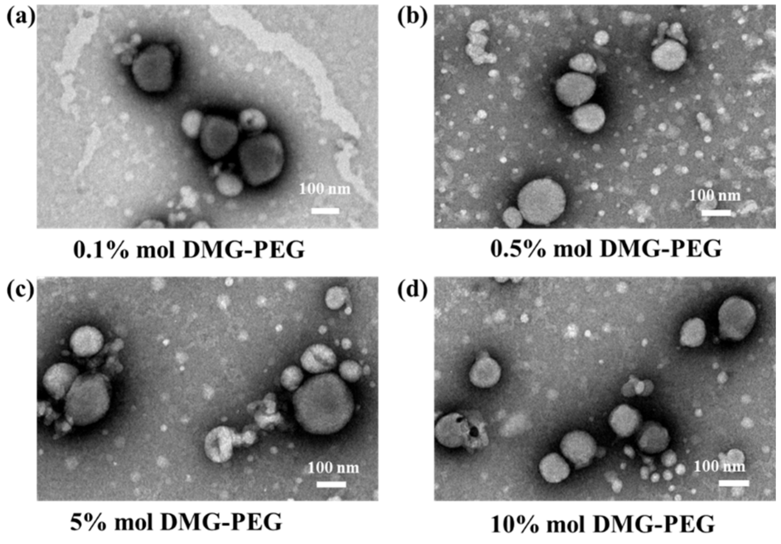


**Figure S2.** Representative images of all formulations with different DMG-PEG contents from 0.1% to 10% (a-d) under TEM.

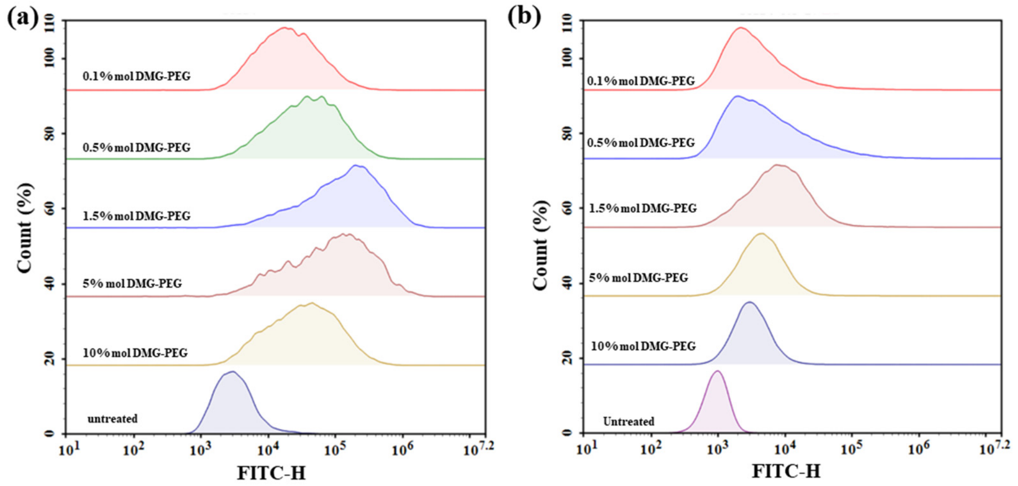


**Figure S3.** Flow cytometry histograms showing fluorescence intensity of LNP/mRNA-transfected cells formulated with varying DMG-PEG contents. (a) HeLa cells. (b) DC 2.4 cells.

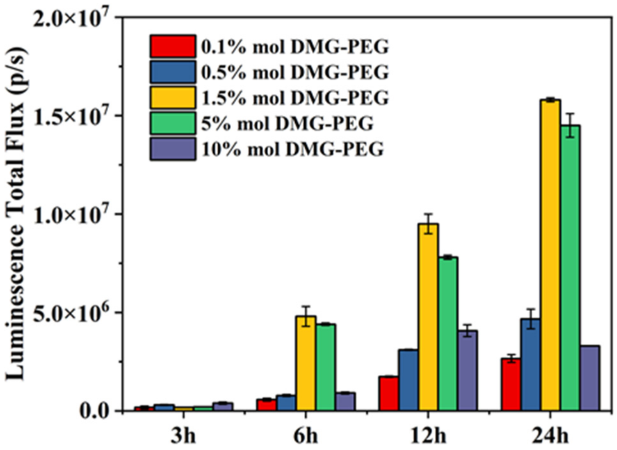


**Figure S4.** mRNA expression kinetics in vitro. HeLa cells were transfected by LNP/mRNA with various DMG-PEG contents for expression of luciferase. The cells were lysed and the luciferase expression was assessed by in vitro Luciferase assay (n = 4).

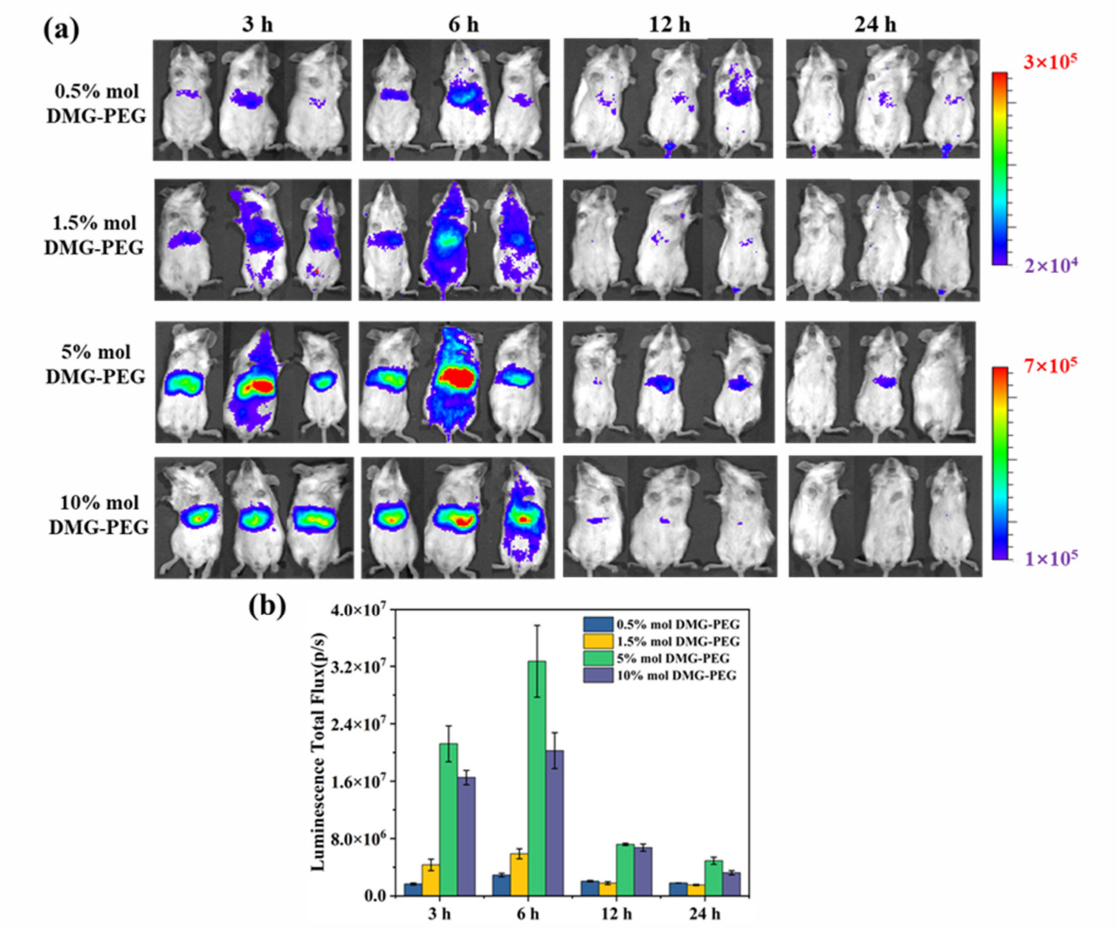


**Figure S5.** mRNA expression kinetics in vivo. (a) In vivo bioluminescence imaging of mice at different time points after i.v. injection of LNP/mRNA with various DMG-PEG contents. (b) Quantification of luciferase expression from IVIS bioimaging (n = 3).

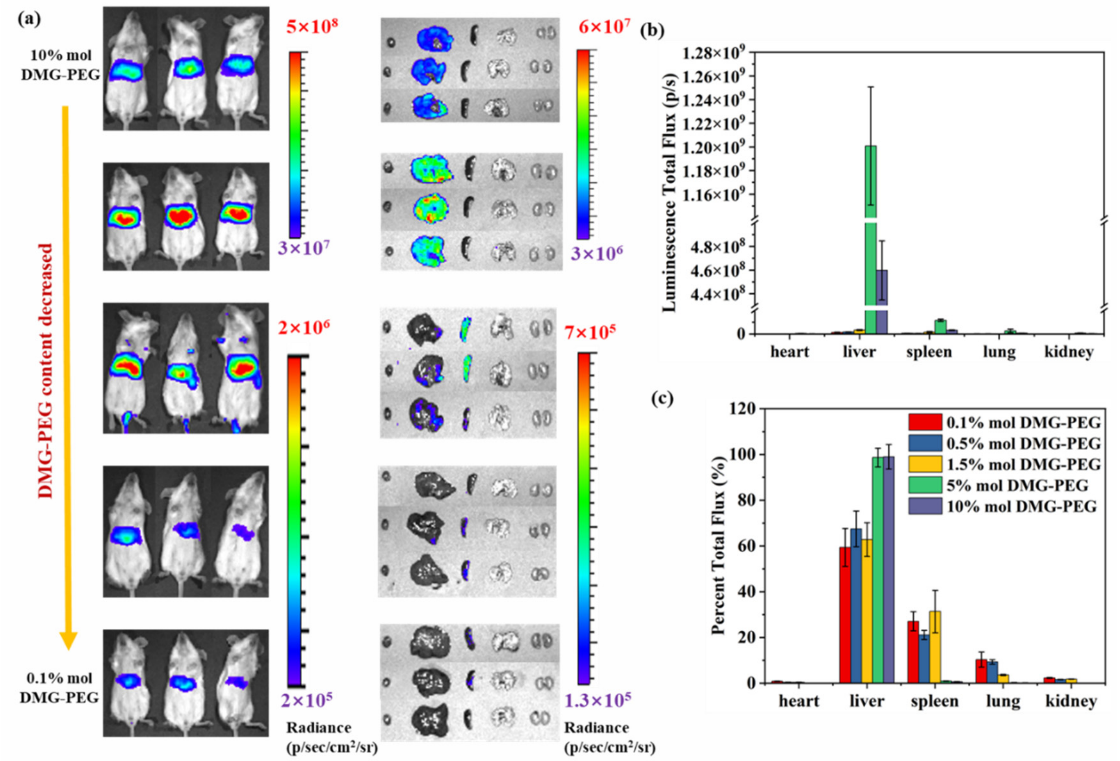


**Figure S6.** In vivo transfection efficiency of ALC-0315-LNP/mluc in mice. Pfizer-LNP formulations with the ionizable lipid (ALC-0315) of 50% and Phospholipid (DSPC) of 10% and lipid-PEG contents from 0.1% to 10% were prepared and i.v. injected similarly as our LNP. (a) In vivo bioluminescence imaging of mice and ex vivo bioluminescence imaging of major organs (heart, liver, spleen, lung and kidneys). (b) Quantitative analysis of luciferase expression in different organs based on bioluminescence intensity (n = 3). (c) Quantification of the percentage of luciferase expression in different organs.
